# Optimization of 3D Surfaces of Dextran with Different Molecule Weights for Real-Time Detection of Biomolecular Interactions by a QCM Biosensor

**DOI:** 10.3390/polym9090409

**Published:** 2017-09-01

**Authors:** Siyu Song, Yuchao Lu, Xueming Li, Shoupeng Cao, Yuxin Pei, Teodor Aastrup, Zhichao Pei

**Affiliations:** 1 Shaanxi Key Laboratory of Natural Products & Chemical Biology, College of Chemistry & Pharmacy, Northwest A&F University, Yangling 712100, China; ssyu137@163.com (S.S.); luyuchao_2006@126.com (Y.L.); lixueming1988@163.com (X.L.); caoshoupeng@nwafu.edu.cn (S.C.); peiyx@nwafu.edu.cn (Y.P.); 2 Attana AB, SE-11419 Stockholm, Sweden; teodor.aastrup@attana.com

**Keywords:** QCM biosensor, 3D dextran surface, surface immobilization capacity, real-time detection, biomolecular interactions

## Abstract

Quartz crystal microbalance (QCM) has been extensively applied in real-time and label-free biomolecular interaction studies. However, the sensitive detection by QCM technology remains challenging, mainly due to the limited surface immobilization capacity. Here, a three-dimensional (3D) carboxymethyl dextran coated gold sensor chip surface was successfully fabricated with dextran of different molecular weight (100, 500 and 2000 kDa, respectively). To evaluate the 3D carboxymethyl dextran surface immobilization capacity, the 3D surface was used for studying antigen–antibody interactions on the QCM biosensor. The results showed that the protein immobilization capacity of the 3D carboxymethyl dextran (2000 kDa) surface exceeded more than 4 times the capacity of the 2D carboxyl surface, and 2 times the capacity of the traditional 3D carboxymethyl dextran (500 kDa) surface. Furthermore, the kinetic and affinity properties of antigen–antibody interactions were performed. Most notably, the optimized 3D carboxymethyl dextran (2000 kDa) surface could be used for small molecule detection, where the binding of biotinylated oligo (0.67 kDa) reached 8.1 Hz. The results confirmed that a 3D carboxymethyl dextran (2000 kDa) surface can be exploited for sensitive detection of low molecular weight analytes, which have great potential applications for characterizing the interactions between small molecule drugs and proteins.

## 1. Introduction

A number of techniques have been employed for the recognition of intermolecular interactions, such as nuclear magnetic resonance (NMR) [1,2,3], X-ray crystallography [4,5], fluorescence spectroscopy [6,7], mass spectrometry [8,9], enzyme-linked lectin assays (ELLAs) [10], ultraviolet (UV)/visible spectroscopy [11,12], surface plasmon resonance (SPR) [13,14,15,16], and quartz crystal microbalance (QCM) [17,18,19]. Several of these tools are often limited in their sensitivity and are difficult to modify for various applications [18]. In contrast, the mass-sensitive biosensor systems, including SPR and QCM, which are known to possess real-time and label-free attributes, have established utility in multiple areas in recent years [20,21]. Both SPR and QCM analytic processes involve attaching one interacting component to the surface of a sensor chip, and then passing a sample containing other interaction partners over the surface. A range of interactions and various binding events occurring on the surface of the sensor chip make the sensor chip an important role in biosensor systems, thus further investigations of optimized sensor chip surfaces leading to versatile performances are of great importance [22].

Surface modification plays a significant role in detecting and characterizing diverse interactions on the sensor chip surface [23]. With different surface modifications, the sensor chips can be tailored to improve adhesion, protein interactions, and cell interactions [24]. There are a wide variety of surface modification approaches suitable for QCM sensor chips. Examples of the sensor chips include polystyrene sensor chips, silicon dioxide sensor chips, gold sensor chips, biotin sensor chips, and carboxyl sensor chips, all of which are 2D sensor chips. In order to achieve high capacity, 3D sensor chips are utilized due to a large amount of available sites. There are several different types of 3D surface reported based on label-free biosensors, including polycarboxylates, surface initiated polymerization, supermolecules, nanoparticles, and vesicles [25,26,27,28,29,30]. For instance, Frasconi and coworkers developed a polycarboxylate-hydrogel-based 3D surface which was used for the real-time detection of cortisol and saliva samples based on SPR technology [25]; Zhu and coworkers reported a 3D SPR imaging sensor chip which was fabricated with carboxylated poly(OEGMA-*co*-HEMA) brushes on the gold surface by surface induced polymerization for diagnosis of Alzheimer’s Disease [26]; Zhao and coworkers reported a 3D-carbene chip for immobilizing various biomolecules by SPR technology [27]; Wang and coworkers demonstrated a 3D supramolecular co-assembly polyrotaxane (PRX) structure surface for detecting peptide interactions by SPR technology, in which more active sites on the surface could increase the immobilization density of molecules [28]; Mao and coworkers reported streptavidin-coated ferrofluid nanoparticles for detection of *Escherichia coli* O157:H7 based on a DNA sensor by QCM technology [29]; Mahon and coworkers demonstrated a 3D glycovesicle surface for detection of the carbohydrate–lectin multivalent biorecognition by QCM technology [30]. The carboxymethyl dextran coated sensor chip surface has attracted our attention for a broader range of applications because it has been used as a template for fabricating 3D surfaces [31].

The 3D carboxymethyl dextran surface with the molecular weight of 500 kDa was first introduced by BIAcore [32], and has been successfully applied in biospecific interaction analysis by SPR technology for a long time [33,34,35,36]. The SPR technology features good sensitivity, for instance, an analyte with low molecular weight of 0.18 kDa could be detected on a carboxymethyl dextran coated surface by SPR [32]. Due to the fact that SPR detection depends on the change in refractive index at the interface of sensor chips, the evanescent wave that is used to measure binding of analyte decays at a distance of about 200 nm away from the sensor chip surface [37]. However, the QCM technology is based on the piezoelectric effect, capable of detecting subtle mass changes in the nanogram range [38], which has been proven to be a powerful and universal tool for the detection of various biospecific interactions [17,39]. The vast majority of reported literatures involving QCM are focused on detections of biomacromolecule interactions such as protein–protein or protein–carbohydrate interactions on 2D surfaces. Few studies reported the application of 3D carboxymethyl dextran surfaces using QCM technology, where their studies focused on biomacromolecule interactions. For instance, Dubiel and coworkers reported the detection and kinetic analysis of Etanercept–protein interactions utilizing a 3D carboxymethyl dextran surface by QCM technology [40]. To the best of our knowledge, there are no reports for the investigation of 3D sensor chip surfaces with dextran of different molecular weights based on QCM technology.

Herein, for the first time, the 3D carboxymethyl dextran sensor chips were successfully fabricated with dextran of different molecular weights (100, 500, and 2000 kDa). To evaluate the chip surface immobilization capacity, the 2D and 3D sensor chip surfaces were used for studying antigen–antibody interactions on the QCM biosensor. Both the immobilization capacity and the binding activity of 2D and 3D surfaces with dextran of different molecular weights were evaluated. Furthermore, the 2D and 3D surfaces with dextran of different molecular weights were also applied in the kinetic study of antigen–antibody interactions. Most notably, the 3D carboxymethyl dextran sensor chip surfaces were used for small molecule detection, which demonstrated the sensitive and label-free detection of a small molecule analyte binding to protein by utilizing a 3D carboxymethyl dextran surface on the QCM biosensor. We believe the optimized 3D carboxymethyl dextran sensor chip could greatly facilitate the application of QCM in characterizing the interactions between small molecule drugs and proteins.

## 2. Materials and Methods

### 2.1. Materials

16-mercaptohexadecan-1-ol was supplied by ProChimia (Sopot, Poland). Tween^®^ 20, streptavidin, epichlorohydrin, diethylene glycol dimethyl ether, bromoacetic acid, sodium hydroxide, Dextran T-100 (*M*w ≈ 100 kDa), Dextran T-2000 (*M*w ≈ 2000 kDa), and 2-[4-(2-hydroxy ethyl)piperazin-1-yl]ethanesulfonic acid (HEPES) were obtained from Sigma-Aldrich (St. Louis, MO, USA). Dextran T-500 (*M*w ≈ 500 kDa) was purchased from Pharmacia (Stockholm, Sweden). 1-ethyl-3-(3dimethyl-aminopropyl) carbodiimine hydrochloride (EDC), *N*-HydroxySuccinimide (NHS), *N*-hydroxysulfosuccinimide (sulfo-NHS), ethanolamine (1 M, pH 8.5), and biotinylated oligo (nicotinamide adenine dinucleotide) were obtained from Attana (Stockholm, Sweden). Myoglobin was obtained from BiosPacific Inc. (Emeryville, CA, USA). Anti-h myoglobin 7005 SPTN-1 was obtained from Medix Biochemica (Espoo, Finland). Other chemicals used were of analytical grade from local suppliers, and used without further purification.

The Attana gold sensor chips and LNB-Carboxyl sensor chips were used in this study. The Attana gold sensor chip was used in basic research and allows modification of the surface for fabrication of the 3D carboxymethyl dextran sensor chips. The LNB-Carboxyl sensor chip features low non-specific binding, thus is one of Attana’s most versatile surfaces for studying molecular interactions.

**Figure 1:**
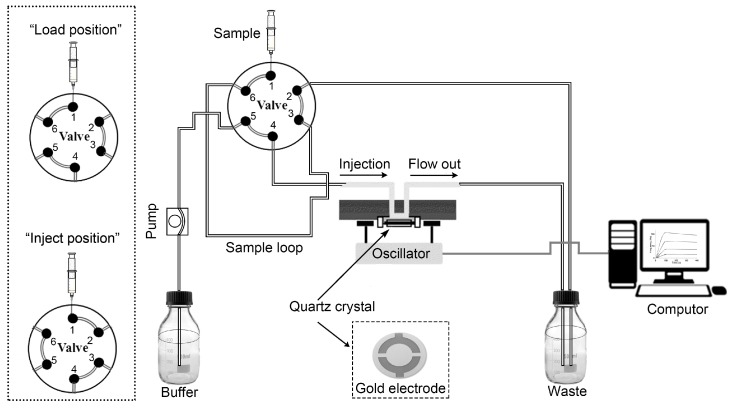
The flow-through system of the Attana QCM biosensor instrument.

To carry out the experiments, the Attana Cell A200 QCM biosensor (Attana AB, Stockholm, Sweden) was used, which was operated in a continuous flow mode (Figure 1). The running buffer is continuously flowing through the flow cell over the sensor surface. When the six-way valve is on “load position”, the connection of six-way valve is 1 to 6, 4 to 5, 2 to 3, as shown in the upper left of Figure 1. At this point, the sample of used ligands or analytes which were prepared in the buffer is injected to the sample loop, and the volume of sample loop is 50 μL. After the sample loop was filled with fluid, the excess fluid overflowed to the waste. And then the valve was switched to “injection position”, the connection of six-way valve is 1 to 2, 3 to 4, 5 to 6, as shown in the bottom left of Figure 1. At this moment, the sample in the sample loop is transported to the sensor surface with the continuous flow [41,42]. Bound samples were released from the surface by injections of low pH buffer. Association/disassociation of samples to the surface was monitored by frequency logging, and the frequency shifts were recorded by the Attester^®^ software (Attana AB). All experiments were carried out at room temperature.

### 2.2. Fabrication of the 3D Carboxymethyl Dextran Sensor Chip with Different Molecular Weight Dextran

The 3D carboxymethyl dextran sensor chip was fabricated according to the protocol published by Löfås and Johnsson [32]. The gold sensor chip surfaces were cleaned in a 1:1:5 (*v/v/v*) solution of H_2_O_2_ (30%), NH_3_ (25%), and ultrapure water for 15 min at 85 °C, and thoroughly rinsed with ultrapure water and ultra-sonicated in 99.5% ethanol for 10 min, and dried under nitrogen flow. The cleaned gold sensor chip surfaces were immersed in a 1 mM solution of HS–(CH_2_)_16_–OH in 99.5% ethanol at room temperature for 16 h, and were rinsed with ethanol and ultrapure water. The surfaces were then reacted with a 0.6 M epichlorohydrin solution in a 1:1 mixture of 0.4 M sodium hydroxide and diglyme on the shaking-table for 4 h at room temperature. After thoroughly washing with ultrapure water three times, the resulting surfaces were immersed in a dextran solution (5.0 g dextran 100 kDa, 500 kDa, and 2000 kDa in 25 mL 0.4 M sodium hydroxide) and incubated on shaking-table for 26 h at room temperature. The surfaces were then rinsed with ultrapure water three times. Further carboxylation on the surfaces was done by reaction with 1 M bromoacetic acid in 2 M sodium hydroxide at room temperature for 16 h. The 3D carboxymethyl dextran matrix surfaces were finally rinsed and then dried with nitrogen directly. The chips were stored in nitrogen, protected from contaminations and dust, at 0–8 °C.

### 2.3. Immobilization of Protein on the Sensor Surface

The 2D carboxyl surface or 3D carboxymethyl dextran surface was first inserted to the QCM sensor system and allowed to stabilize at a flow rate of 100 μL/min in HEPES-buffered saline running buffer (10 mM HEPES, 150 mM NaCl, 0.005% Tween^®^ 20, pH 7.4). When the baseline was stable (baseline drift <0.2 Hz/min), the flow rate was lowered to 5 μL/min.

To immobilize Anti-h myoglobin 7005, the chip surface was activated twice with a 1:1 mixture of 0.4 M EDC and 0.1 M NHS or sulfo-NHS for a period of 10 min each. Anti-h myoglobin 7005 dissolved in 10 mM acetic acid buffer, pH 4.5 at a concentration of 50 μg/mL, was thereafter injected three times over the activated chip surface. To deactivate any remaining NHS esters, two injections of 1 M ethanolamine pH 8.5 were performed for a period of 10 min each.

### 2.4. Protein–Protein Interactions and Kinetic Characterization with QCM

To study the interaction of Anti-h myoglobin 7005 with its interacting protein, the flow rate was set to 25 µL/min. The interacting protein myoglobin was diluted in running buffer at a series of concentration and injected over the chip surface. Regeneration was performed by 0.1 M glycine pH 1.5 to remove the interacting protein. The binding of the two proteins was monitored for a period of 85 s for association and 300 s for dissociation. The frequency shifts (Δ*f*) were recorded with the Attester software and could be loaded directly from Attester Evaluation software. The data was then analyzed by the software ClampXP, which is a biosensor kinetic data analysis program developed to interpret interaction kinetics by fitting data to appropriate models. ClampXP performs a number of interaction analysis with the fit of a theoretical 1:1 interaction model that are influenced by mass transport limitation [43]. The fitted data sets were used to interpret the kinetics of macromolecular interactions, such as the association rate constant: *k*_on_, the dissociation rate constant *k*_off_, and the affinity constant *K*_D_, which characterize the kinetics of the interaction.

### 2.5. Detection of Low Molecular Weight Oligo by QCM

The 3D carboxymethyl dextran sensor chip surface was first inserted to the QCM instrument and allowed to stabilize at a flow rate of 100 μL/min in HEPES-buffered saline running buffer. When the baseline was stable (baseline drift <0.2 Hz/min), the flow rate was set to 5 μL/min. The sensor chip surface was activated twice with a reagent mixture of EDC and NHS at final concentrations of 0.2 M and 0.05 M twice for a period of 10 min each. Subsequent to surface activation, streptavidin diluted in 10 mM acetic acid buffer, pH 4.5 at a concentration of 50 μg/mL, was injected three times over the activated surface. To deactivate remaining NHS esters on the surface, two injections of 1 M ethanolamine pH 8.5 was performed with a contact time of 10 min each. The surface was allowed to stabilize until drift was less than 1 Hz over 10 min. HEPES-buffered saline running buffer was injected over the surface as blank for 600 s, waiting at least 600 s after injection. Then the biotinylated oligo was dissolved in running buffer at 10 μM and injected over the surface for a contact time of 600 s.

## 3. Results and Discussion

**Figure 2:**
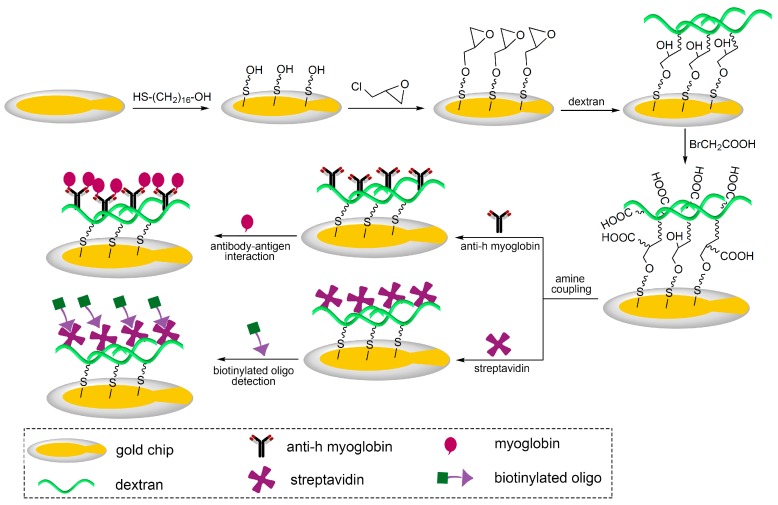
Schematic illustration of 3D carboxymethyl dextran sensor chip surfaces for real-time detection of biomolecular interactions by a QCM biosensor.

The 3D carboxymethyl dextran sensor chip surfaces were fabricated with dextran of different molecular weights as shown in Figure 2. In this work, the interaction between the immobilized protein and its receptor was monitored using a QCM biosensor. The immobilization capacity and the binding activity of the 3D surfaces (100, 500, and 2000 kDa) were compared, and the 3D carboxymethyl dextran surface with the molecular weight of 2000 kDa resulted in the highest sensitivity for enhanced surface capacity. Most notably, the 3D carboxymethyl dextran (2000 kDa) surface provided the QCM biosensor accessibility to acquire adequate response signals even probing detections of low molecule weight samples.

### 3.1. Comparison of Immobilization and Binding Capacity on 2D and 3D Surfaces

**Figure 3:**
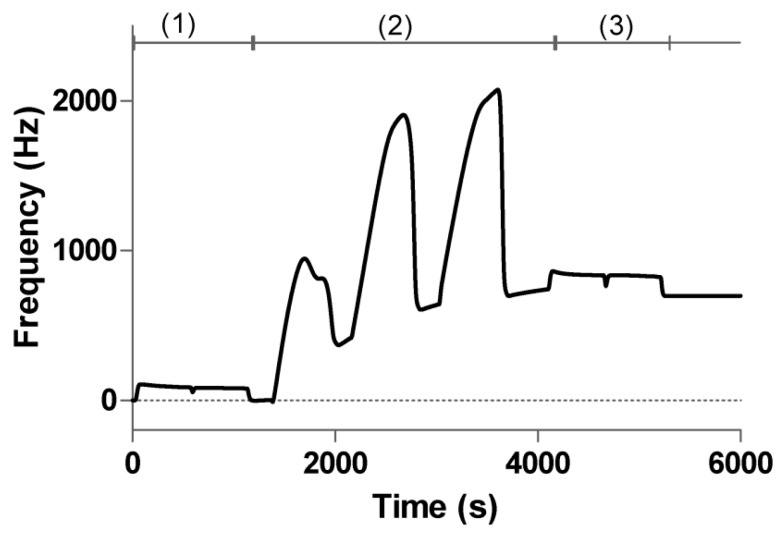
Typical sensorgram of Anti-h myoglobin 7005 immobilization via amine coupling on 3D carboxymethyl dextran surface (2000 kDa). (1) activation by EDC and NHS; (2) coupling of Anti-h myoglobin 7005; (3) deactivation with ethanolamine.

In our experiments, the comparisons of the immobilization of Anti-h myoglobin protein via amine coupling on 2D and 3D carboxymethyl dextran surfaces were carried out by QCM. For the 2D chip surface, the carboxyl surface was activated with EDC/sulfo–NHS [44], and for the 3D surface, the surface was activated with EDC/NHS [45]. Subsequently, Anti-h myoglobin 7005 was dissolved in acetic acid buffer (pH 4.5) at 50 μg/mL for three injections through the activated sensor chip surface at 5 μL/min. Remaining active groups on the surface were deactivated by 1 M ethanolamine at pH 8.5. The typical example of the immobilization sensorgram on 3D carboxymethyl dextran surface (2000 kDa) is shown in Figure 3. To test the binding capacity, the interaction protein myoglobin was diluted in the running buffer of 4 µg/mL at 25 μL/min. Compared to the 2D surface, the flexible dextran chain illustrates higher immobilization and binding capacity. In particular, the 3D carboxymethyl dextran surface of 2000 kDa, exhibited the greatest immobilization and binding capacity among the three 3D surfaces modified with different molecular weight dextran.

**Figure 4:**
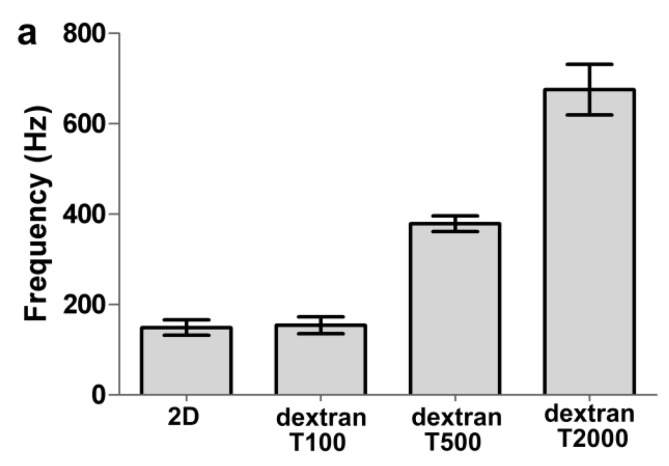

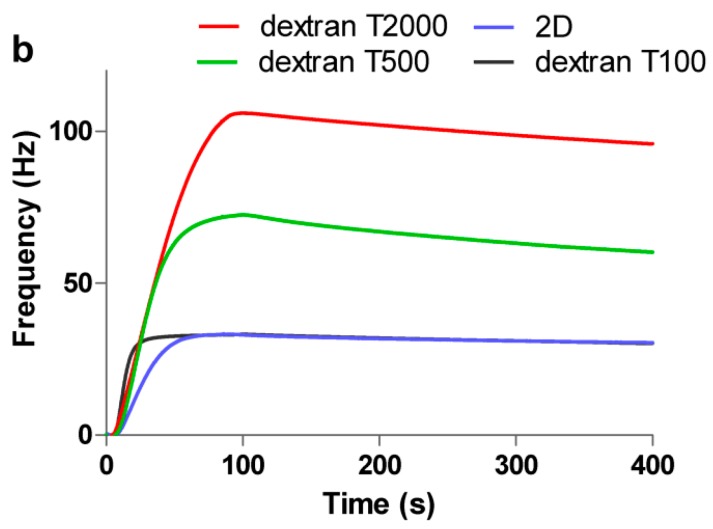
(**a**) The frequency response of Anti-h myoglobin 7005 to immobilized, Anti-h myoglobin 7005 at 50 μg/mL was introduced onto the different surfaces; (**b**) Comparison of the binding of myoglobin on different surfaces, myoglobin at 4 μg/mL was injected to the 2D and 3D surfaces respectively. All experiments were performed under the same conditions.

As shown in Figure 4a, the frequency shift (149 Hz) was obtained from immobilization of Anti-h myo 7005 on the 2D surface, and the frequency shifts of 155, 375, and 697 Hz were obtained from immobilization of Anti-h myo 7005 on the 3D carboxymethyl dextran (100, 500, and 2000 kDa) surface. The frequency illustrated the binding capacity of the immobilized protein, that is the higher the frequency, the greater number of events detected. The protein immobilization capacity of the 3D (100 kDa) surface was nearly the same as the 2D surface. Interestingly, the protein immobilization capacity of the 3D (500 kDa) surface exceeded more than 2 times the capacity of the 2D chip surface. Significantly, the protein immobilization capacity of the 3D (2000 kDa) surface exceeded more than 4 times the capacity of the 2D surface. Such a remarkable enhancement in immobilization capacity allowed the QCM to detect adequate response signals during the binding of myoglobin. The signals of myoglobin binding on 2D and 3D surfaces are shown in Figure 4b. The 3D surface (2000 kDa) captured about 100 Hz of the myoglobin, which is approximately 3 times than that of the 2D surface, and the 3D surface (500 kDa) captured about 60 Hz of the myoglobin, which is twice as much as that obtained on the 2D surface.

### 3.2. Interaction and Kinetic Studies

**Figure 5:**
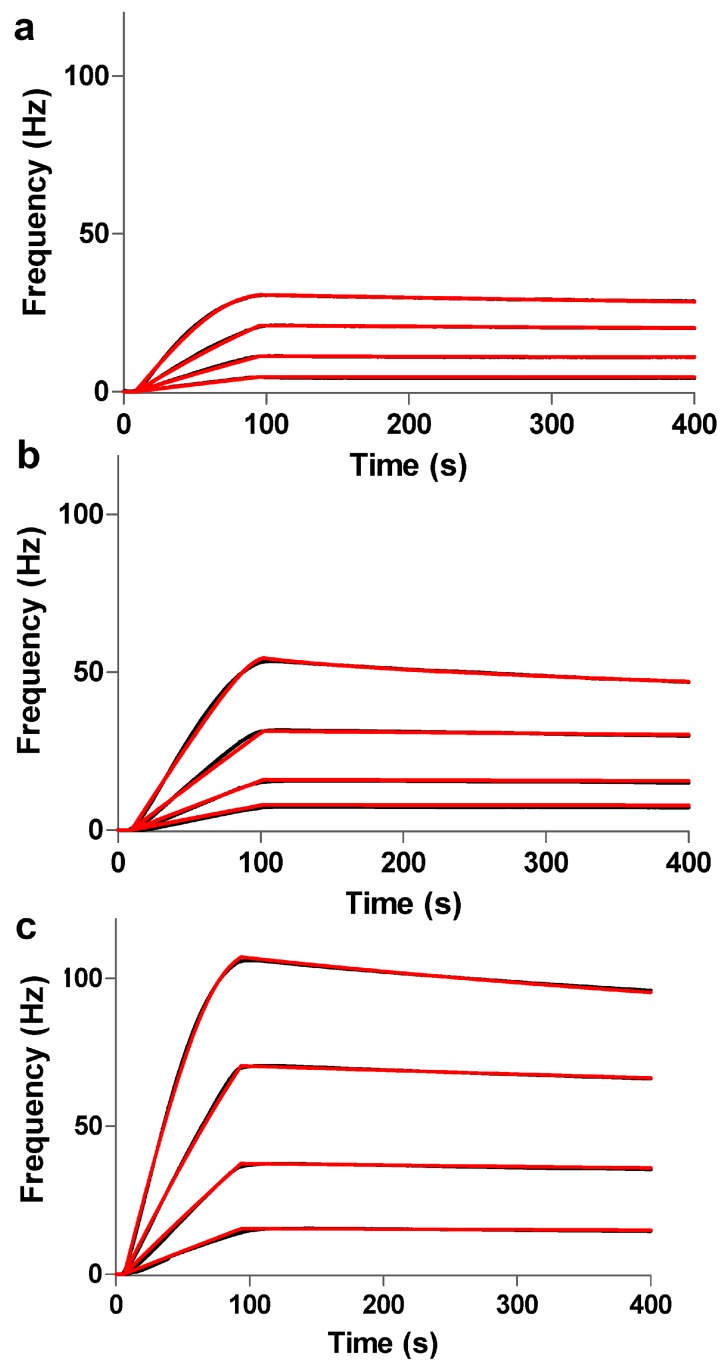
(**a**) Kinetic evaluation of the interaction on the 2D surface, myoglobin at 0.03, 0.06, 0.12, and 0.24 µM, was injected over the surface, respectively; (**b**) Kinetic evaluation on the 3D carboxymethyl dextran (500 kDa) surface, myoglobin at 0.03, 0.06, 0.12, and 0.24 µM, was injected over the surface, respectively; (**c**) Kinetic evaluation on the 3D carboxymethyl dextran (2000 kDa) surface, myoglobin at 0.03, 0.06, 0.12, and 0.24 µM, was injected over the surface, respectively. In Figure 5a–c, the measured responses are presented in black lines, the theoretical 1:1 fit generated by the ClampXP software are overlaid in red lines.

The interactions between Anti-h myoglobin 7005 and myoglobin were evaluated using the Attana Cell A200 QCM biosensor. To study the kinetic and affinity properties of the interaction between the captured Anti-h myoglobin 7005 and its interacting protein myoglobin on the different surfaces, myoglobin was diluted in the running buffer and then injected over the surface at a series of concentrations from 0.03 µM to 0.24 µM. The interaction between Anti-h myoglobin 7005 and myoglobin was monitored for 85 s for association and for 300 s for dissociation. The surface was regenerated via 0.1 M glycine (pH 1.5) injection, which removed the interacting protein myoglobin after each cycle, and the measured responses are presented in Figure 5 along with the theoretical 1:1 fit produced by the ClampXP software. The frequency shift (149 Hz) was obtained from immobilization of Anti-h myo 7005 on the 2D surface, and myoglobin at 0.03, 0.06, 0.12, and 0.24 µM were injected over the surface, giving the resultant *k*_on_ = 8.31 × 10^5^ M^−1^·s^−1^, *k*_o__ff_ = 9.71 × 10^−4^ s^−1^ and *K*_D_ = 1.17 nM, which is displayed in Figure 5a. The immobilization frequency of Anti-h myo 7005 was recorded with maximum frequency shifts of 375 Hz on the 3D carboxymethyl dextran (500 kDa) surface, and myoglobin at 0.03, 0.06, 0.12, and 0.24 µM were injected over the surface, giving the resultant *k*_on_ = 5.80 × 10^5^ M^−1^·s^−1^, *k*_o__ff_ = 7.16 × 10^−4^ s^−1^ and *K*_D_ =1.23 nM, which is displayed in Figure 5b. The frequency shift (697 Hz) was obtained from immobilization of Anti-h myo 7005 on the 3D carboxymethyl dextran (2000 kDa) surface, and myoglobin at 0.03, 0.06, 0.12, and 0.24 µM were injected over the surface, giving the resultant *k*_on_ = 4.17 × 10^5^ M^−1^·s^−1^, *k*_o__ff_ = 5.21 × 10^−4^ s^−1^ and *K*_D_ = 1.25 nM, which is displayed in Figure 5c. Furthermore, the 3D surface also produced different binding characteristics compared to the 2D surface, revealing that both the association and dissociation rates on the 3D surface are slower than that on the 2D surface. We hypothesize that the lower association rate on the 3D surface is mainly due to mass transfer limitation and steric hindrance effects as the protein required a finite time to fully penetrate the dextran matrix. The lower dissociation rate may be due to the larger number of binding sites on the 3D surface [31].

### 3.3. Regeneration and Stability of the Sensing Surface

**Figure 6:**
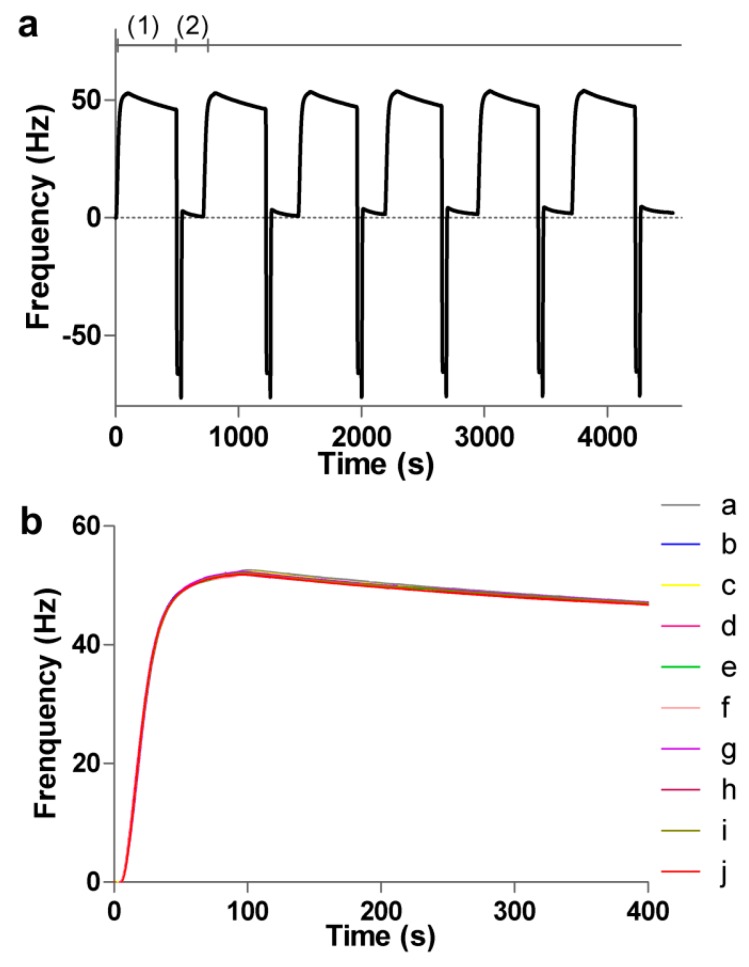
(**a**) Binding of myoglobin to the immobilized 3D carboxymethyl dextran (2000 kDa) surface and subsequent release of the bound myoglobin via injection of low pH buffer (0.1 M glycine, pH 1.5). (1) binding of myoglobin; (2) regeneration with 0.1 M glycine. The 3D surface was completely regenerated after one injection of the glycine solution for 30 s; (**b**) the reproducibility of the surface was examined by running 10 repeats of the myoglobin.

Surface regeneration is one of the significant factors in the study of biomolecule interactions. Successful surface regeneration for cyclic use would make the sensor chips more cost effective and the experiments less time-consuming. The protein Anti-h myo 7005 diluted in acetic acid buffer pH 4.5 at a concentration of 50 μg/mL was conjugated to the 3D carboxymethyl dextran (2000 kDa) surface by amine coupling, then myoglobin dissolved in the running buffer was injected over the surface. Surface regeneration was performed by removing the bound protein for the next analysis cycle. The surface was successfully regenerated when all of the remaining bound protein had been desorbed from the surface. A typical sensorgram is displayed in Figure 6a, in which two injections of myoglobin at 2 μg/mL were performed successively within the regeneration step, including one injection of 0.1 M glycine pH 1.5 for 30 s, in between. The reproducibility of the surface was examined by running 10 repeats of the myoglobin injection at the same concentration and monitoring the binding curve to verify their similarity. The results are presented in Figure 6b. The standard deviation of the baseline value does not exceed 0.4% of the immobilization level of myoglobin after 10 cycles. The 3D carboxymethyl dextran surface (2000 kDa) was capable of being used for several cycles of analyses without any significant change in baseline. All the measurements were performed at a flow rate of 25 μL/min. The results indicated good reversibility and reproducibility of the 3D carboxymethyl dextran (2000 kDa) surface.

### 3.4. Evaluating Sensitivity of the Surface

**Figure 7:**
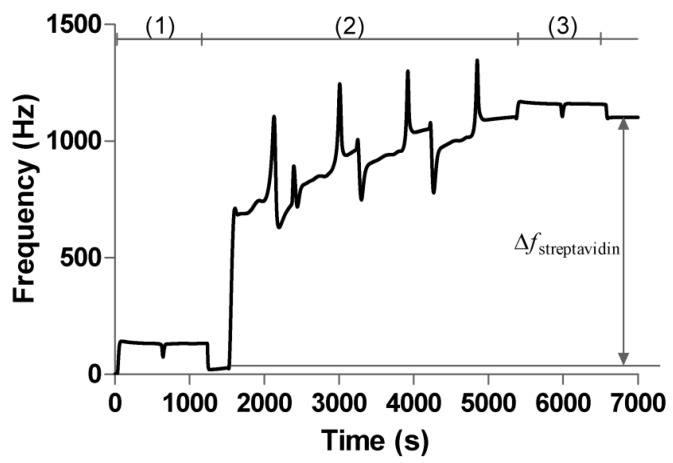
Procedure for immobilizing streptavidin via amine coupling on 3D carboxymethyl dextran (2000 kDa) surface; (1) activation by EDC and NHS; (2) coupling of streptavidin; (3) deactivation with ethanolamine.

**Figure 8:**
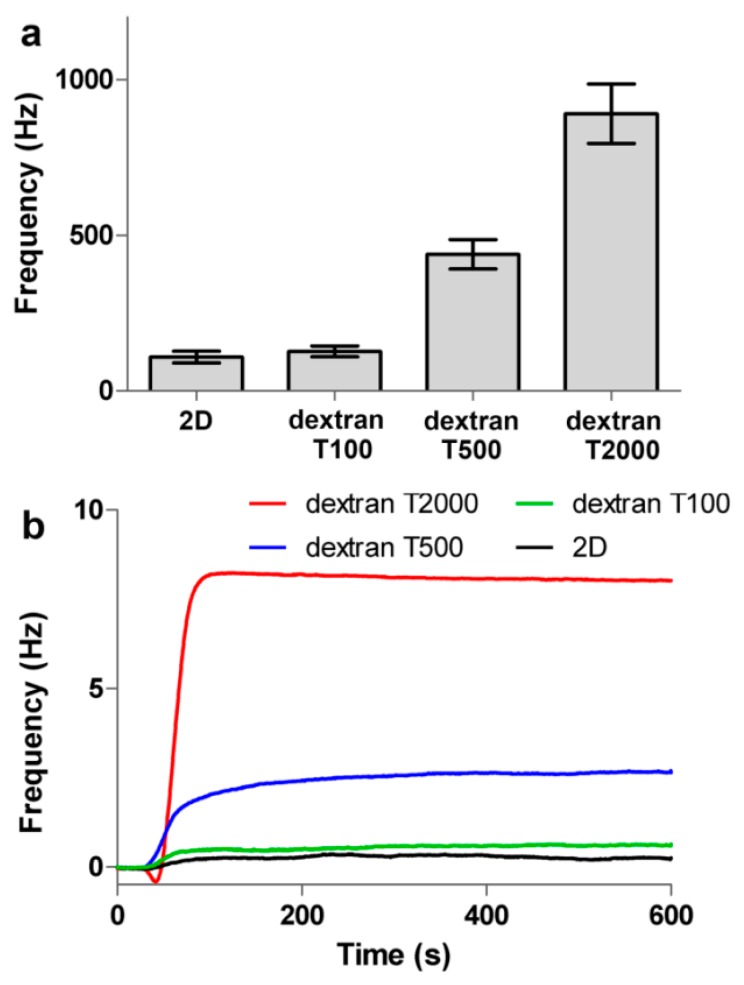
(**a**) The frequency response of streptavidin immobilized onto different surfaces; (**b**) Comparison of the binding of biotinylated oligo on different surfaces, biotinylated oligo at a concentration of 10 μM was injected to the 2D and 3D surfaces, respectively. The measured responses on 3D carboxymethyl dextran (2000 kDa) surface are presented as a red line, and the measured responses on 3D carboxymethyl dextran (500 kDa) surface are shown as the blue line. All experiments were performed under the same conditions.

In order to investigate the influence of the dextran with different molecular weights on the sensitivity of sensor chip surface, the 2D carboxyl surface and 3D carboxymethyl dextran surfaces (100, 500, and 2000 kDa) were all used for small molecule detection. For small molecule detection using QCM, sufficient protein immobilization on the surface should be considered. Thus, after activation of the surface by 0.2 M EDC and 0.05 M NHS for a period of 20 min at 5 μL/min, streptavidin diluted in acetic acid buffer pH 4.5 at a concentration of 50 μg/mL was conjugated to the sensor chip surface by amine coupling. The typical example of the immobilization sensorgram on 3D carboxymethyl dextran surface (2000 kDa) is shown in Figure 7. Compared to the 2D surface, the 3D surface exhibited increased immobilization capacity. As shown in Figure 8a, the frequency shift (110 Hz) was obtained from immobilization of streptavidin on the 2D surface, and the frequency shifts of 125, 430, and 900 Hz were obtained from immobilization of streptavidin on the 3D carboxymethyl dextran (100, 500, and 2000 kDa) surface, respectively. Then, the biotinylated oligo was dissolved in running buffer at a concentration of 10 μM and injected over the surface for a contact time of 600 s, and the running buffer was injected over 600 s over the surface as a reference before every injection of the biotinylated oligo. While no response was observed on 2D surface and 3D carboxymethyl dextran (100 kDa) surface, 2.5 Hz of the biotinylated oligo was bound to the streptavidin on the 3D carboxymethyl dextran (500 kDa) sensor chip surface, and 8.1 Hz of the biotinylated oligo was bound to the streptavidin on the 3D carboxymethyl dextran (2000 kDa) sensor chip surface, which are presented in Figure 8b. The result confirmed that 2D and 3D carboxymethyl dextran (100 kDa) surfaces were not capable of detecting small molecular binding events, while the 3D carboxymethyl dextran (500 and 2000 kDa) allowed QCM to detect adequate response signals when probing small molecule binding events.

## 4. Conclusions

In this paper, 3D carboxymethyl dextran surfaces were successfully fabricated with dextran of different molecular weight (100, 500, and 2000 kDa). To evaluate their surface immobilization capacity, each surface was used for studying antigen–antibody interactions on the QCM biosensor. Advantageous protein immobilization capacity of the 3D (2000 kDa) surface has been confirmed, showing that the immobilization capacity was improved more than 4 times compared to a 2D surface, and more than 2 times compared to a traditional 3D carboxymethyl dextran (500 kDa) surface. Furthermore, interactions between proteins were detected on the 2D surface and different 3D surfaces, providing more in-depth information about binding kinetics and affinity. The optimized 3D surface (2000 kDa) is able to completely regenerate for several cycles, providing stable immobilization and good reproducibility. Most notably, the 3D carboxymethyl dextran (2000 kDa) surface could be used for small molecule detection, where the binding signal of biotinylated oligo reached 8.1 Hz, while no response was observed on 2D surface. As we know, the detection of small molecular weight analytes with QCM technology is a restriction, and this study will enhance the application in various fields using QCM technology. These inspiring results confirm that the 3D carboxymethyl dextran (2000 kDa) surface demonstrated great potential as a promising platform in characterizing the interactions between small molecule drugs and proteins, which may have further impacts on the discovery of drugs.
